# The nature of metallophilic interactions in closed-shell d^8^–d^8^ metal complexes†

**DOI:** 10.1039/d4cp00250d

**Published:** 2024-07-18

**Authors:** Lucas de Azevedo Santos, Timon Wagner, Klaas Visscher, Jörn Nitsch, F. Matthias Bickelhaupt, Célia Fonseca Guerra

**Affiliations:** a Department of Chemistry and Pharmaceutical Sciences, AIMMS, Vrije Universiteit Amsterdam De Boelelaan 1108 1081 HZ Amsterdam The Netherlands c.fonsecaguerra@vu.nl f.m.bickelhaupt@vu.nl https://www.theochem.nl; b Institute for Molecules and Materials, Radboud University Heyendaalseweg 135 6525 AJ Nijmegen The Netherlands; c Department of Chemical Sciences, University of Johannesburg Auckland Park Johannesburg 2006 South Africa

## Abstract

We have quantum chemically analyzed the closed-shell d^8^–d^8^ metallophilic interaction in dimers of square planar [M(CO)_2_X_2_] complexes (M = Ni, Pd, Pt; X = Cl, Br, I) using dispersion-corrected density functional theory at ZORA-BLYP-D3(BJ)/TZ2P level of theory. Our purpose is to reveal the nature of the [X_2_(CO)_2_M]⋯[M(CO)_2_X_2_] bonding mechanism by analyzing trends upon variations in M and X. Our analyses reveal that the formation of the [M(CO)_2_X_2_]_2_ dimers is favored by an increasingly stabilizing electrostatic interaction when the M increases in size and by more stabilizing dispersion interactions promoted by the larger X. In addition, there is an overlooked covalent component stemming from metal–metal and ligand–ligand donor–acceptor interactions. Thus, at variance with the currently accepted picture, the d^8^–d^8^ metallophilicity is attractive, and the formation of [M(CO)_2_X_2_]_2_ dimers is not a purely dispersion-driven phenomenon.

## Abstract 2

d^8^–d^8^ square planar metal-complex dimers bind through electrostatic and dispersion interactions, augmented by HOMO–LUMO interactions between the metal centers (important for larger metals) and between the ligands (important for larger ligands).

## Graphical abstract

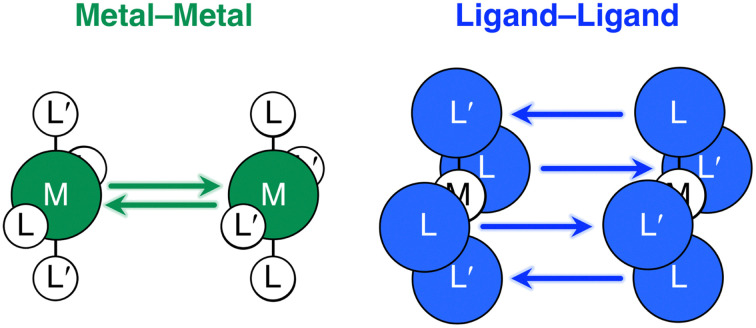

## 1. Introduction

Metallophilicity is the manifestation of a net attractive intra- or intermolecular interaction between metal centers (M), yielding metal–metal-bonded complexes.^1^ This metal–metal interaction causes the spontaneous association, *i.e.*, self-assembly, of metal complexes forming aggregates with luminescence properties^2^ and medicinal applications.^3^ Earlier work by Hoffman *et al.* showed that the closed-shell metallophilic interactions between linear d^10^ metal complexes are covalent due to the donor–acceptor interactions between the filled *n*d_*Z*^2^_-type HOMO on one M and the (*n* + 1)s-type LUMO on the other M.^4^ However, Brands *et al.* showed that the donor–acceptor interactions cannot overcome the destabilizing steric Pauli repulsion between the two metal centers, and the metallophilic interactions are only attractive due to the additional stabilizing electrostatic interactions.^5^ In general, the closed-shell d^10^–d^10^ metalllophilic interactions have a similar mechanistic picture to other well-known intermolecular interactions, such as hydrogen bonds.^6^

Nonetheless, alternative models have been used to explain metallophilicity. For example, Pyykkö and coworkers concluded that the d^10^–d^10^ Au^+^⋯Au^+^ attraction in staggered dimers of linear AuPH_3_Cl complexes is a dispersion-driven phenomenon, whereas the ligand–ligand repulsion hampers the formation of parallel dimers.^7^ This has been further supported by Chen *et al.* who found that π–π interactions between face-to-face, eclipsed ligands are repulsive.^8^ On the other hand, Che *et al.* attributed the formation of d^8^–d^8^ and d^10^–d^10^ metal-complexes dimers to stabilizing ligand–ligand dispersion interactions.^9^

**Scheme 1:**
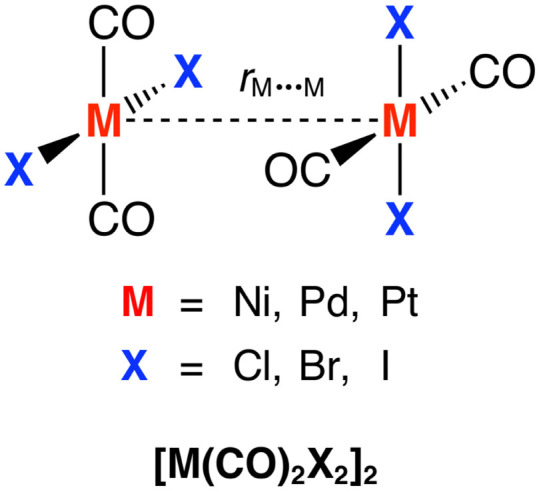
The studied [M(CO)_2_X_2_]_2_ dimers.

In the present study, we have quantum chemically investigated the bonding mechanism of eclipsed closed-shell d^8^–d^8^ metal complexes, using [M(CO)_2_X_2_]_2_ dimers (M = Ni, Pd, Pt; X = Cl, Br, I) as model systems (Scheme 1). As will become clear in the following sections, our bonding analyses reveal that the stability of the [M(CO)_2_X_2_]_2_ dimers stems from significantly attractive electrostatic interactions, on top of dispersion interactions promoted by the ligands. This closed-shell d^8^–d^8^ metal–metal interaction is, therefore, similar to that between d^10^ metal centers, that is, it is also partially covalent in nature and, thus, not a pure dispersion-driven phenomenon.

## 2. Computational methods

### 2.1. Computational details

All calculations were carried out using the Amsterdam Modeling Suite (AMS) 2020.101 program.^10^ All stationary points and energies were obtained using relativistic, dispersion-corrected density functional theory at ZORA-BLYP-D3(BJ)/TZ2P. This approach has been benchmarked in previous work for bond energies of metal–metal complexes using CCSD(T)/CBS with mean absolute error of 0.7 kcal mol^−1^.^5^ This approach comprises the BLYP level of the generalized gradient approximation (GGA); exchange functional developed by Becke (B), and the GGA correlation functional developed by Lee, Yang, and Parr (LYP)^11^ (see Tables S4 and S5 in the ESI,† for the Cartesian coordinates). The DFT-D3(BJ) method developed by Grimme and coworkers,^12^ which contains the damping function proposed by Becke and Johnson,^13^ is used to describe non-local dispersion interactions. Scalar relativistic effects are accounted for using the zeroth-order regular approximation (ZORA).^14^ Molecular orbitals (MO) were expanded in a large, uncontracted set of Slater-type orbitals (STOs) containing diffuse functions: TZ2P.^15^ The basis set is of triple-ζ quality augmented with polarization functions, *i.e.*, one 3d and one 4f set on C, O, Cl; one 4d and one 4f set on Br; one 5d and one 4f set on I; one 4f set on Ni; one 5p and one 4f set on Pd; one 6p and one 5f set on Pt. All electrons were included in the variational process, *i.e.*, no frozen core approximation was applied. The accuracies of both the fitting scheme and the integration grid (Becke grid) were set to ‘EXCELLENT’. The formation of *trans*-[M(CO)_2_X_2_] monomers and *trans*-[M(CO)_2_X_2_]_2_ dimers (M = Ni, Pd, Pt; X = Cl, Br, I) is, in most cases, more favorable than the formation of *cis*-[M(CO)_2_X_2_] monomers and *cis*-[M(CO)_2_X_2_]_2_ dimers (see Table S3 and Fig. S4 for bond energies and geometries, ESI†). The [Ni(CO)_2_Br_2_], [Ni(CO)_2_I_2_], and [Pd(CO)_2_I_2_] monomers, and their respective dimers, are the only exceptions in which the *cis* forms are more favorable (Table S3, ESI†). Thus, in this study, we always refer to the *trans*-[M(CO)_2_X_2_]_2_ dimers. We have found here that the global-minimum structures of the [M(CO)_2_X_2_]_2_ dimers are in the staggered conformation by performing a fully relaxed rotation around the M⋯M bond, in which the X–M⋯M–X dihedral angle is varied from 0° to 90° (see Fig. S5 and S6 in the ESI†). All optimized structures were confirmed to be true minima through vibrational analysis (no imaginary frequencies).

### 2.2. Bond analyses

Insight into the bonding mechanism is obtained by analyzing the potential energy curves for the formation of the [M(CO)_2_X_2_]_2_ dimers (M = Ni, Pd, Pt; X = Cl, Br, I). The analyses are done by varying the M⋯M bond distance within the range of 2.5 Å to 4.5 Å, starting from the equilibrium geometry of the complex (*r*_M⋯M,eq_) while keeping all other geometrical parameters frozen.

The interaction between [X_2_(CO)_2_M] and [M(CO)_2_X_2_] is analyzed using the activation strain model,^16^ which is a fragment-based approach to understanding the energy profile associated with a chemical process in terms of the original reactants. Thus, the total bond energy Δ*E* is decomposed along the M⋯M bond distance *r*_M⋯M_ (or just at one point along *r*_M⋯M_) into the strain energy Δ*E*_strain_(*r*_M⋯M_), which is associated with the geometrical deformation of the individual reactants as the process takes place, plus the actual interaction energy Δ*E*_int_(*r*_M⋯M_) between the deformed reactants [eqn (1)].

1 Δ*E*(*r*_M⋯M_) = Δ*E*_strain_(*r*_M⋯M_) + Δ*E*_int_(*r*_M⋯M_)

In the equilibrium geometry, that is, for *r*_M⋯M_ = *r*_M⋯M,eq_, this yields an expression for the bond energy Δ*E*(*r*_M⋯M,eq_) = Δ*E* = Δ*E*_strain_ + Δ*E*_int_. The interaction energy Δ*E*_int_(*r*_M⋯M_) between the deformed reactants is further analyzed in the conceptual framework provided by the quantitative Kohn–Sham MO model.^17^ To this end, it is decomposed into physically meaningful terms [eqn (2)] using a quantitative energy decomposition analysis (EDA) as implemented in ADF.^17^

2 Δ*E*_int_(*r*_M⋯M_) = Δ*V*_elstat_(*r*_M⋯M_) + Δ*E*_Pauli_(*r*_M⋯M_) + Δ*E*_oi_(*r*_M⋯M_) + Δ*E*_disp_(*r*_M⋯M_)

The usually attractive term Δ*V*_elstat_ corresponds to the classical Coulomb interaction between the unperturbed charge distributions of the deformed reactants and has four components: (i) the electron–electron repulsion between the electron densities of [X_2_(CO)_2_M] and [M(CO)_2_X_2_]; (ii) the nuclei–electron attraction between the nuclei of [X_2_(CO)_2_M] and the electron density of [M(CO)_2_X_2_]; (iii) the electron–nuclei attraction between the electron density of [X_2_(CO)_2_M] and the nuclei of [M(CO)_2_X_2_]; and (iv) the nuclei–nuclei repulsion between the nuclei of [X_2_(CO)_2_M] and [M(CO)_2_X_2_].^17^

The Pauli repulsion energy (Δ*E*_Pauli_) comprises the destabilizing interactions between the fully occupied orbitals on either fragment and arises from the antisymmetrization of the Hartree wavefunction due to the Pauli principle. The orbital-interaction energy (Δ*E*_oi_) accounts for charge transfer, that is, the interaction between occupied orbitals of one fragment with unoccupied orbitals of the other fragment, including the interactions of the highest occupied and lowest unoccupied MOs (HOMO–LUMO), and polarization, that is, empty–occupied orbital mixing on one fragment, due to the presence of another fragment. The dispersion energy Δ*E*_disp_ accounts for the dispersion corrections as introduced by Grimme *et al.*^12^ To facilitate the analyses, the ASM and EDA were performed using the PyFrag 2019 program.^18^

## 3. Results and discussion

### 3.1. Bond strength and structure

**Fig. 1:**
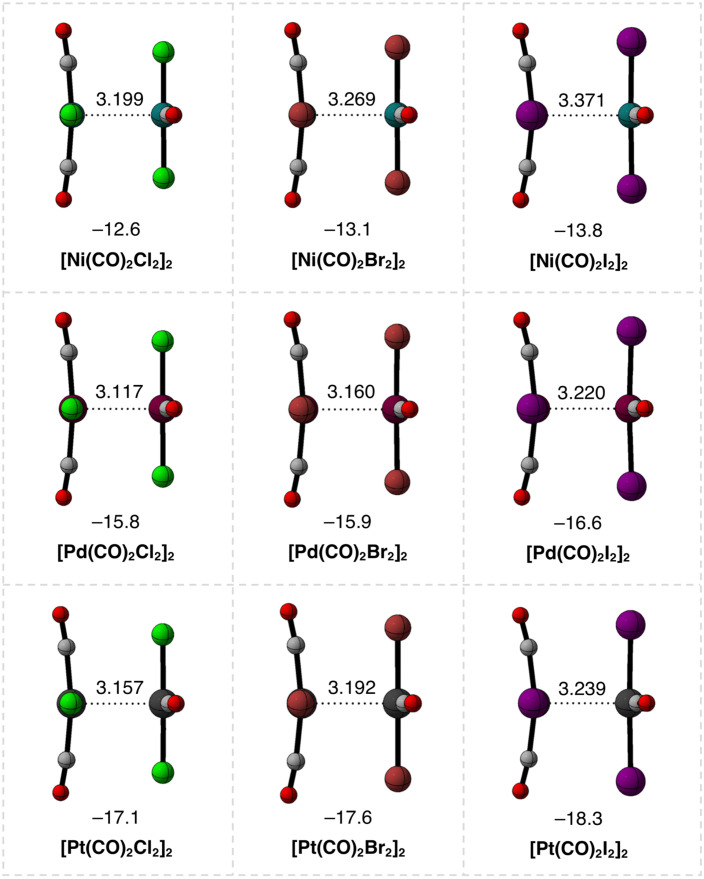
Equilibrium geometries (in Å) and electronic bond energies (in kcal mol^−1^) of the [M(CO)_2_X_2_]_2_ dimers (M = Ni, Pd, Pt; X = Cl, Br, I). Computed at ZORA-BLYP-D3(BJ)/TZ2P.

Equilibrium geometries, dimerization energies (Δ*E*), and the M⋯M bond distances (*r*_M⋯M_) of the stable *C*_S_ symmetrical [M(CO)_2_X_2_]_2_ dimers (M = Ni, Pd, Pt; X = Cl, Br, I) calculated at the ZORA-BLYP-D3(BJ)/TZ2P level of theory are shown in Fig. 1. Upon the formation of the studied dimers, we have identified two main features: (i) the stability of the dimers significantly increases, and the M⋯M bond distance slightly shortens for heavier M; and (ii) the dimers are only slightly stronger but longer for heavier X ligands. For example, along the series from [Ni(CO)_2_Br_2_]_2_ to [Pt(CO)_2_Br_2_]_2_, Δ*E* becomes more stabilizing from −13.1 kcal mol^−1^ to −17.6 kcal mol^−1^ and *r*_M⋯M_ decreases from 3.269 Å to 3.192 Å (Fig. 1). Along the series from [Pd(CO)_2_Cl_2_]_2_ to [Pd(CO)_2_I_2_]_2_, Δ*E* varies only from −15.8 kcal mol^−1^ to −16.6 kcal mol^−1^ and *r*_M⋯M_ increases from 3.117 Å to 3.220 Å (Fig. 1).

Next, we performed our activation strain analysis^16^ (ASA) to decompose the bond energies (Δ*E*) into the strain (Δ*E*_strain_) and interaction (Δ*E*_int_) energies (see Computational methods), and the results are shown in Table 1. The trends in the stability of the [M(CO)_2_X_2_]_2_ dimers along M = Ni, Pd, Pt and X = Cl, Br, I are dominated by the stronger Δ*E*_int_, whereas the weaker Δ*E*_strain_ varies only slightly along the same series. For example, along the series from [Ni(CO)_2_Br_2_]_2_ to [Pt(CO)_2_Br_2_]_2_, Δ*E*_int_ becomes more stabilizing from −14.0 kcal mol^−1^ to −18.9 kcal mol^−1^ and Δ*E*_strain_ becomes slightly more destabilizing from 0.9 kcal mol^−1^ to 1.3 kcal mol^−1^ (Table 1). Along the series from [Pd(CO)_2_Cl_2_]_2_ to [Pd(CO)_2_I_2_]_2_, Δ*E*_int_ becomes more stabilizing from −16.6 kcal mol^−1^ to −17.9 kcal mol^−1^ and Δ*E*_strain_ becomes slightly more destabilizing from 0.8 kcal mol^−1^ to 1.3 kcal mol^−1^ (Table 1). Note that the impact on Δ*E*_int_ and, thus, on the stability of the [M(CO)_2_X_2_]_2_ dimers when varying M is larger than when varying X. As will become clear in the next sections, this is because of the difference in nature between the metal–metal interactions and those involving the ligands. This conclusion emerges from understanding the physical nature behind the observed trends in Δ*E*_int_ when the metal center M varies along Ni, Pd, and Pt and the ligands X vary along Cl, Br, and I.

**Table 1:** Activation strain and energy decomposition analyses (in kcal mol^−1^) for the formation of the [M(CO)_2_X_2_] dimers (M = Ni, Pd, Pt; X = Cl, Br, I) in their equilibrium geometries (in Å). Computed at ZORA-BLYP-D3(BJ)/TZ2P

M	X	*r*_M⋯M_	Δ*E*	Δ*E*_strain_	Δ*E*_int_	Δ*V*_elstat_	Δ*E*_Pauli_	Δ*E*_oi_	Δ*E*_disp_
Ni	Cl	3.199	−12.6	0.6	−13.2	−12.3	20.3	−7.6	−13.6
	Br	3.269	−13.1	0.9	−14.0	−14.8	21.3	−8.0	−15.4
	I	3.371	−13.8	0.9	−14.7	−11.6	22.7	−7.7	−18.1

Pd	Cl	3.117	−15.8	0.8	−16.6	−24.1	34.8	−11.4	−15.8
	Br	3.160	−15.9	1.3	−17.2	−23.1	35.8	−12.0	−17.9
	I	3.220	−16.6	1.3	−17.9	−22.3	37.1	−12.0	−20.8

Pt	Cl	3.157	−17.1	0.9	−18.0	−28.0	39.5	−12.9	−16.6
	Br	3.192	−17.6	1.3	−18.9	−27.3	41.0	−13.8	−18.9
	I	3.239	−18.3	1.8	−20.1	−26.8	43.2	−14.2	−22.1

### 3.2. Variation of M

To understand the origin of the increased stabilization of the [M(CO)_2_X_2_]_2_ dimers upon varying M along Ni, Pd, and Pt, we further decomposed the Δ*E*_int_ into physically meaningful terms, namely the electrostatic interactions (Δ*V*_elstat_), steric Pauli repulsion (Δ*E*_Pauli_), orbital interactions (Δ*E*_oi_), and dispersion energy (Δ*E*_disp_) using our energy decomposition analysis^17^ (EDA; see Computational methods), and the results are shown in Table 1. Our analyses reveal that, for the smaller Ni, we find that Δ*E*_disp_ is the largest attractive term in [X_2_(CO)_2_M]⋯[M(CO)_2_X_2_]. However, as M increases in size, Δ*V*_elstat_ and Δ*E*_oi_ become significantly more stabilizing whereas Δ*E*_disp_ varies much less, which causes Δ*V*_elstat_ to dominate and become the most attractive term for Pd and Pt. For example, from [Ni(CO)_2_Br_2_]_2_ to [Pt(CO)_2_Br_2_]_2_, Δ*E*_disp_ varies only from −15.4 kcal mol^−1^ to −18.9 kcal mol^−1^, whereas Δ*V*_elstat_ and Δ*E*_oi_ become significantly more stabilizing from −14.8 kcal mol^−1^ to −27.3 kcal mol^−1^ and from −8.0 kcal mol^−1^ to −13.8 kcal mol^−1^, respectively (Table 1).

**Fig. 2:**
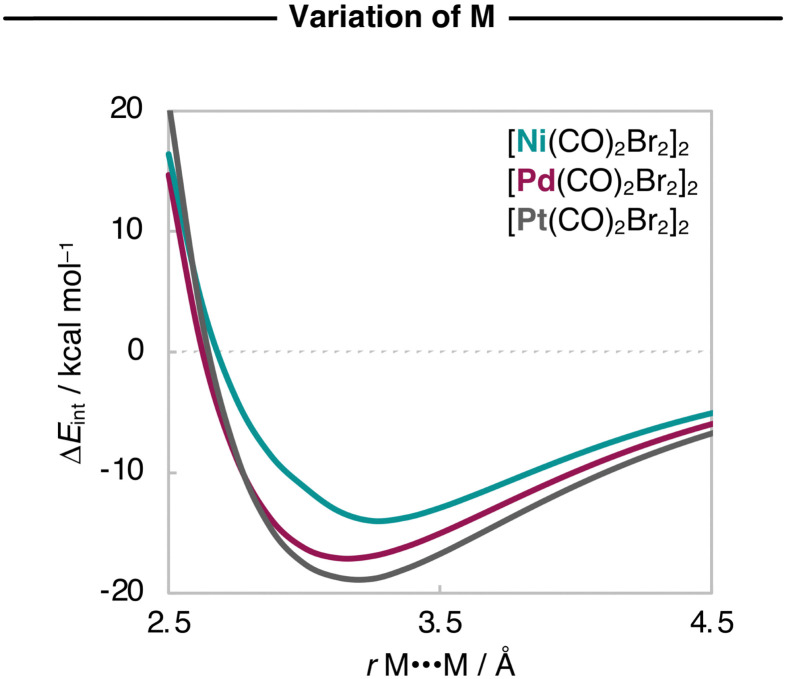
Interaction energies (in kcal mol^−1^) as a function of the M⋯M distance (in Å) starting from the equilibrium geometries of the representative [M(CO)_2_Br_2_]_2_ dimers (M = Ni, Pd, Pt), while the geometry of the monomers is kept frozen. Computed at ZORA-BLYP-D3(BJ)/TZ2P.

For a consistent comparison, we extend our analysis to the entire reaction coordinate, as a function of the M⋯M bond distance (*r*_M⋯M_). Since Δ*E*_strain_ is small and the Δ*E* is dominated by Δ*E*_int_, the analyses were done while the geometries of the fragments were kept frozen to that of the equilibrium geometries of the [M(CO)_2_X_2_]_2_ dimers. The resulting interaction energy curves Δ*E*_int_(*r*_M⋯M_) for the representative [Ni(CO)_2_Br_2_]_2_, [Pd(CO)_2_Br_2_]_2_, and [Pt(CO)_2_Br_2_]_2_ series are graphically shown in Fig. 2. Herein, the Δ*E*_int_(*r*_M⋯M_) curves also become more stabilizing and the energy minimum is shifted towards shorter *r*_M⋯M_ as M varies along Ni, Pd, and Pt.

**Fig. 3:**
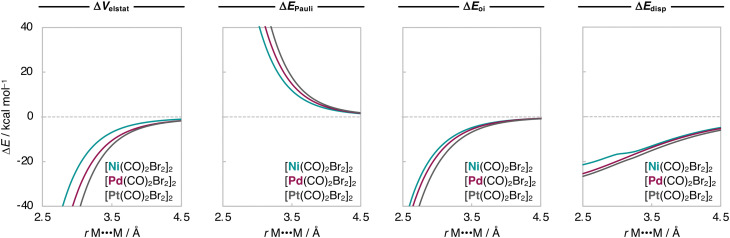
Energy decomposition analysis (in kcal mol^−1^) as a function of the M⋯M distance (in Å) for the representative [M(CO)_2_Br_2_]_2_ dimers (M = Ni, Pd, Pt), while the geometry of the monomers is kept frozen. Computed at ZORA-BLYP-D3(BJ)/TZ2P.

**Fig. 4:**
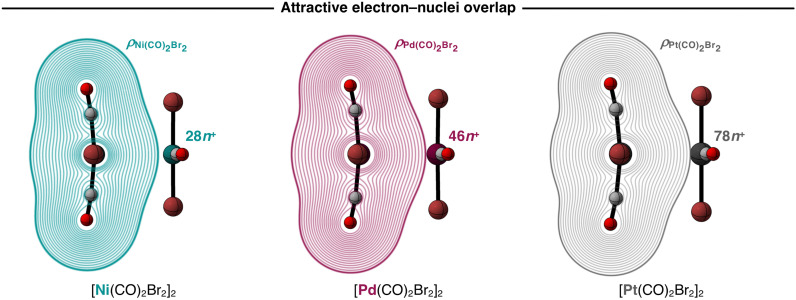
Attractive overlap between the negatively charged electron densities and the positively charged nuclei (density contours from 0.0001 to 0.5000 a.u.) for the representative [M(CO)_2_Br_2_]_2_ dimers (M = Ni, Pd, Pt) at a consistent M⋯M bond distance (*r*_M⋯M_ = 3.5 Å). Computed at ZORA-BLYP-D3(BJ)/TZ2P.

Next, we analyze the EDA terms as a function of *r*_M⋯M_ and the resulting diagrams for the representative [Ni(CO)_2_Br_2_]_2_, [Pd(CO)_2_Br_2_]_2_, and [Pt(CO)_2_Br_2_]_2_ series are graphically shown in Fig. 3. Our findings show that the increased stability of the [M(CO)_2_Br_2_]_2_ dimers as M varies along Ni, Pd, Pt is due to a greater electrostatic attraction between the monomers, as the electrostatic interaction curves Δ*V*_elstat_(*r*_M⋯M_) become significantly more stabilizing along the same series (Fig. 3). This is because the [X_2_(CO)_2_M]⋯[M(CO)_2_X_2_] electrostatic attraction is largely affected by the charge distributions around the metal centers within the monomers. In essence, there is a more effective electron–nuclei overlap as the electron density (*ρ*) of [M(CO)_2_Br_2_]_2_ becomes more diffuse, and the nucleus of the metal center increases in size (*i.e.*, has more protons) along the same series. For example, at the same point of the reaction coordinate, *e.g.*, *r*_M⋯M_ = 3.5 Å, the negatively charged *ρ* of one fragment is more diffuse around the metal center and extends further towards the nucleus of the metal center of the other fragment, which becomes more positively charged as M varies along Ni, Pd, and Pt (see Fig. 4). As the two monomers approach each other, this attractive electron–nuclei overlap more quickly increases for heavier M and, consequently, the slope of the descending Δ*V*_elstat_(*r*_M⋯M_) curves increases along Ni, Pd, and Pt, shifting the equilibrium geometries of the [M(CO)_2_Br_2_]_2_ dimers to a shorter *r*_M⋯M_ as M varies along the same series.

**Scheme 2:**
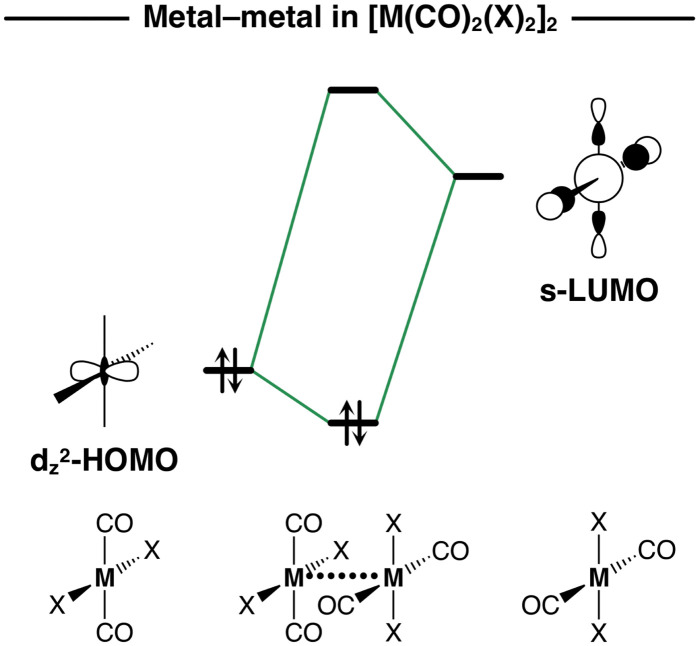
Schematic molecular orbital diagram of the metal–metal donor–acceptor interactions in [M(CO)_2_X_2_]_2_ dimers. The DFT orbitals are shown in Fig. S3 of the ESI.†

Together with Δ*V*_elstat_(*r*_M⋯M_), the orbital interactions Δ*E*_oi_(*r*_M⋯M_) and the dispersion energy curves Δ*E*_disp_(*r*_M⋯M_) follow the same trend as Δ*E*_int_(*r*_M⋯M_) and become more stabilizing along M = Ni, Pd, Pt (Fig. 3). This effect is more pronounced in Δ*E*_oi_(*r*_M⋯M_) which arises, in part, from the donation of charge from the metal d_*Z*^2^_-type HOMO of one M(CO)_2_X_2_ monomer into the metal s-type LUMO of the other monomer and will be explained in detail later (see Scheme 2). The Δ*E*_oi_(*r*_M⋯M_) curves are weakly attractive at longer *r*_M⋯M_ and, as soon as the d_*Z*^2^_-HOMO and s-LUMO start to overlap at shorter *r*_M⋯M_, they become significantly stabilizing (Fig. 3). The Δ*E*_disp_(*r*_M⋯M_) curves, on the other hand, are relatively strong already at longer *r*_M⋯M_, but neither become much more stabilizing at shorter *r*_M⋯M_ nor significantly vary along M = Ni, Pd, Pt. In other words, the Δ*E*_disp_ term significantly contributes to the stability of the [M(CO)_2_X_2_]_2_ dimers but is almost insensitive to variations of the metal centers.

### 3.3. Variation of X

To understand why the Δ*E*_int_, and thus the [M(CO)_2_X_2_]_2_ dimers, becomes slightly more stable upon varying X along Cl, Br, and I, we first analyze the EDA terms at the equilibrium geometries. Contrary to the variation along M = Ni, Pd, Pt, our analyses reveal that Δ*E*_disp_ becomes more stabilizing, whereas Δ*V*_elstat_ and Δ*E*_oi_ do not significantly change when X varies along Cl, Br, and I. For example, from [Pd(CO)_2_Cl_2_]_2_ to [Pd(CO)_2_I_2_]_2_, Δ*E*_disp_ becomes more stabilizing from −15.8 kcal mol^−1^ to −20.8 kcal mol^−1^, whereas Δ*V*_elstat_ and Δ*E*_oi_ vary from −24.1 kcal mol^−1^ to −22.3 kcal mol^−1^ and −11.4 kcal mol^−1^ to −12.0 kcal mol^−1^, respectively (Table 1).

**Fig. 5:**
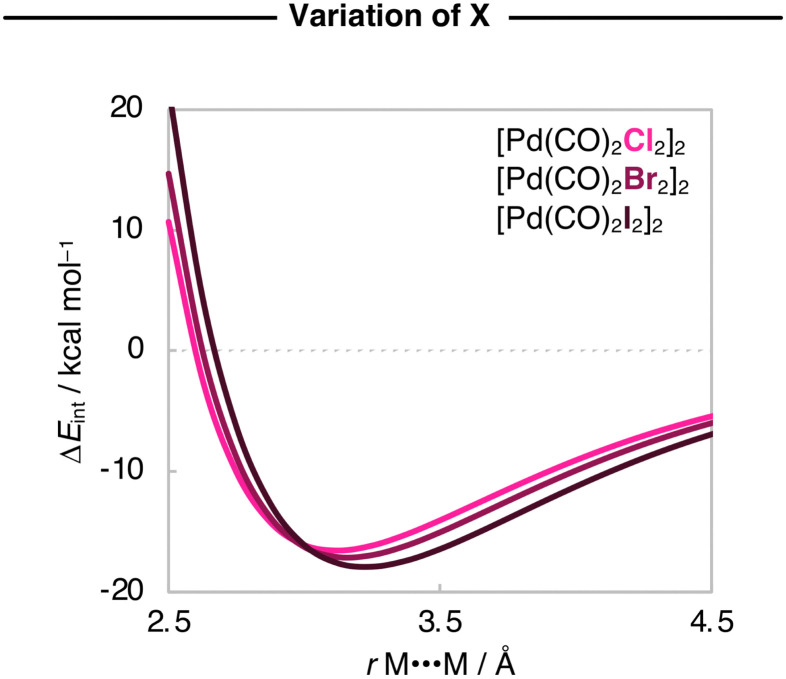
Interaction energies (in kcal mol^−1^) as a function of the M⋯M distance (in Å) starting from the equilibrium geometries of the representative [Pd(CO)_2_X_2_]_2_ dimers (X = Cl, Br, I), while the geometry of the monomers is kept frozen. Computed at ZORA-BLYP-D3(BJ)/TZ2P.

**Fig. 6:**
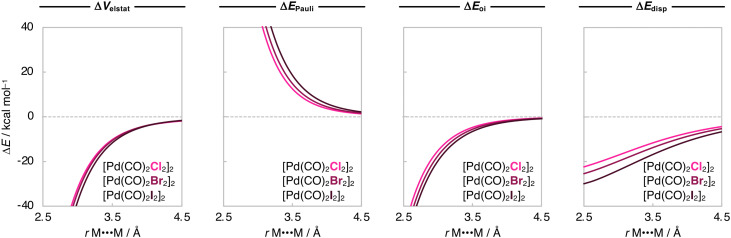
Energy decomposition analysis (in kcal mol^−1^) as a function of the M⋯M distance (in Å) for the representative [Pd(CO)_2_X_2_]_2_ dimers (X = Cl, Br, I), while the geometry of the monomers is kept frozen. Computed at ZORA-BLYP-D3(BJ)/TZ2P.

Next, we analyze the Δ*E*_int_ and the EDA terms as a function of *r*_M⋯M_, and the resulting diagrams for the representative [Pd(CO)_2_Cl_2_]_2_, [Pd(CO)_2_Br_2_]_2_, and [Pd(CO)_2_I_2_]_2_ series are graphically shown in Fig. 5 and 6. The Δ*E*_int_(*r*_M⋯M_) curves also become more stabilizing, and the energy minimum is shifted towards longer *r*_M⋯M_ as X varies along Cl, Br, and I. Our analyses show that the increased stability of the [Pd(CO)_2_X_2_]_2_ dimers as X varies along Cl, Br, and I is due to a greater attractive dispersion interaction promoted by the ligands as they increase in size along the same series. Note that the Δ*E*_disp_(*r*_M⋯M_) curves become significantly more stabilizing along [Pd(CO)_2_Cl_2_]_2_, [Pd(CO)_2_Br_2_]_2_, and [Pd(CO)_2_I_2_]_2_ (Fig. 6). Nevertheless, the equilibrium *r*_M⋯M_ slightly expands because the Pauli repulsion curves Δ*E*_Pauli_(*r*_M⋯M_) are steeper than the Δ*E*_disp_(*r*_M⋯M_) curves and push the equilibrium *r*_M⋯M_ to longer values as X varies along Cl, Br, and I (Fig. 6).

The Δ*V*_elstat_(*r*_M⋯M_) curves are almost insensitive to variations of X in the [Pd(CO)_2_X_2_]_2_ dimers and become only slightly more stabilizing along X = Cl, Br, I (Fig. 6). This is because, as aforementioned, the attractive electron–nuclei overlap occurs mainly at the M⋯M bond region and does not significantly change as the X ligands increase in size along X = Cl, Br, I. Instead, the increased size of X causes the *ρ* of the monomers to extend further along the CO⋯X region, where there is not a pronounced electron–nuclei overlap (see Fig. S1, ESI†). Consequently, the electrostatic attraction between the monomers in the [M(CO)_2_X_2_]_2_ only marginally increases when X varies along Cl, Br, and I.

**Scheme 3:**
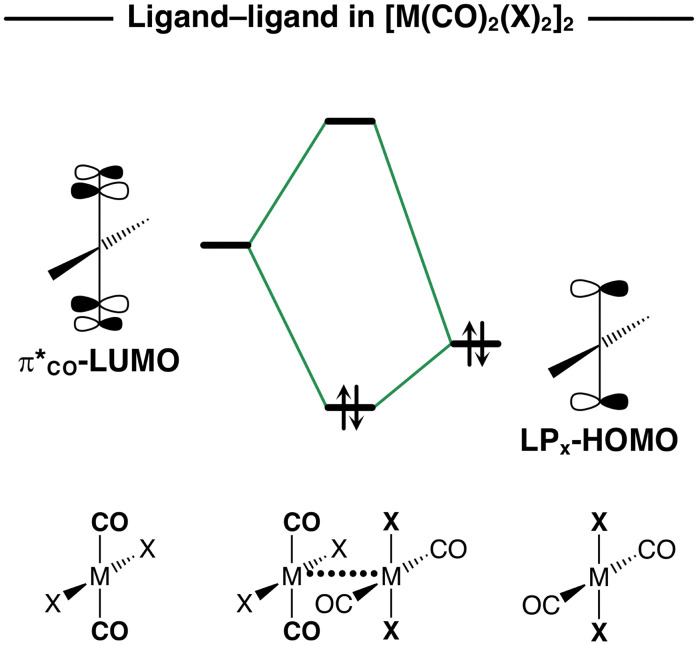
Schematic molecular orbital diagram of the ligand–ligand donor–acceptor interactions in [M(CO)_2_X_2_]_2_ dimers. The DFT orbitals are shown in Fig. S3 of the ESI.†

Our analyses along variations of M and X have revealed two important interactions for the bonding mechanism and, thus, stability of the [M(CO)_2_X_2_]_2_ dimers. On the one hand, there are electrostatic interactions that increase in relevance as the metal becomes bigger. On the other hand, there are attractive dispersion interactions stemming from the ligands. Despite this difference in nature between the metal–metal and ligand–ligand interactions, they also share some similarities. Similar to the trend upon varying M along Ni, Pd, and Pt, the Δ*E*_oi_(*r*_M⋯M_) curves also become more stabilizing when X varies along Cl, Br, and I in the [Pd(CO)_2_X_2_]_2_ dimers (Fig. 6). This is due to the stabilizing ligand–ligand donor–acceptor interaction of the C=O π*-type (π*_CO_) LUMO of one monomer with the X lone-pair-type (LP_X_) HOMO of the other monomer (see Scheme 3). In the following section, we explain why the Δ*E*_oi_(*r*_M⋯M_) curves become more stabilizing along M = Ni, Pd, Pt and X = Cl, Br, I in the [M(CO)_2_X_2_]_2_ dimers.

### 3.4. Metal–metal *versus* ligand–ligand donor–acceptor interactions

In previous sections, we showed that the stability of the [M(CO)_2_X_2_]_2_ dimers is mainly governed by the electrostatic attraction, which becomes more important for larger M and dispersive interactions promoted by the ligands. However, both metal–metal and ligand–ligand interactions have a covalent component stemming from donor–acceptor interactions that has been hitherto overlooked. Note that the Δ*E*_oi_ between the monomers in [M(CO)_2_X_2_]_2_ dimers, which accounts for donor–acceptor interactions, becomes more stabilizing along M = Ni, Pd, Pt and X = Cl, Br, I (*vide supra*). Next, we explain the physical nature behind these trends.

The donor–acceptor interactions in the [M(CO)_2_X_2_]_2_ dimers comprise of two main components, namely, the metal–metal d_*Z*^2^_-HOMO–s-LUMO and the ligand–ligand π*_CO_-LUMO–LP_X_-HOMO interactions (see Schemes 2 and 3). The relevance of a donor–acceptor interaction can be estimated by the magnitude of its orbital stabilization, which is proportional to its HOMO–LUMO overlap squared (*S*^2^) divided by its respective orbital energy gap (Δ*ε*) [see eqn (3)]. For a consistent comparison, we report these values for the metal–metal and ligand–ligand donor–acceptor interactions at *r*_M⋯M_ = 3.5 for all [M(CO)_2_X_2_]_2_ dimers in Table 2.

3 Δ*E*_oi_ ∝ *S*^2^/Δ*ε*

**Table 2:** Orbital interaction energies (in kcal mol^−1^), orbital overlap, energy gap (Δ*ε*, in eV), and orbital stabilization for the metal–metal and ligand–ligand donor–acceptor interactions in the [M(CO)_2_X_2_]_2_ dimers (M = Ni, Pd, Pt; X = Cl, Br, I) at a consistent M⋯M bond distance (*r*_M⋯M_ = 3.5 Å). Computed at ZORA-BLYP-D3(BJ)/TZ2P

M	X	Δ*E*_oi_	Metal–metal				Ligand–ligand			
			*S*	Δ*ε*^a^	10^3^×*S*^2^/Δ*ε*	%^b^	*S*	Δ*ε*^a^	10^3^×*S*^2^/Δ*ε*	%^b^
Ni	Cl	−3.8	0.11	8.2	1.5	53	0.07	3.8	1.3	47
	Br	−4.9	0.09	7.8	1.0	36	0.08	3.5	1.8	64
	I	−5.8	0.08	7.3	0.9	25	0.09	3.1	2.6	75

Pd	Cl	−4.7	0.18	8.4	3.9	67	0.08	3.4	1.9	33
	Br	−5.6	0.17	8.1	3.6	58	0.09	3.1	2.6	42
	I	−6.5	0.16	7.7	3.3	47	0.10	2.7	3.7	53

Pt	Cl	−5.8	0.20	8.2	4.9	67	0.09	3.3	2.5	33
	Br	−6.8	0.18	8.1	4.0	55	0.10	3.0	3.3	45
	I	−8.0	0.17	7.8	3.7	44	0.11	2.6	4.7	56

a See HOMO and LUMO energies in Tables S1 and S2 (ESI).

b Contribution of the associated orbital stabilization to the total metal–metal + ligand–ligand orbital stabilization.

**Fig. 7:**
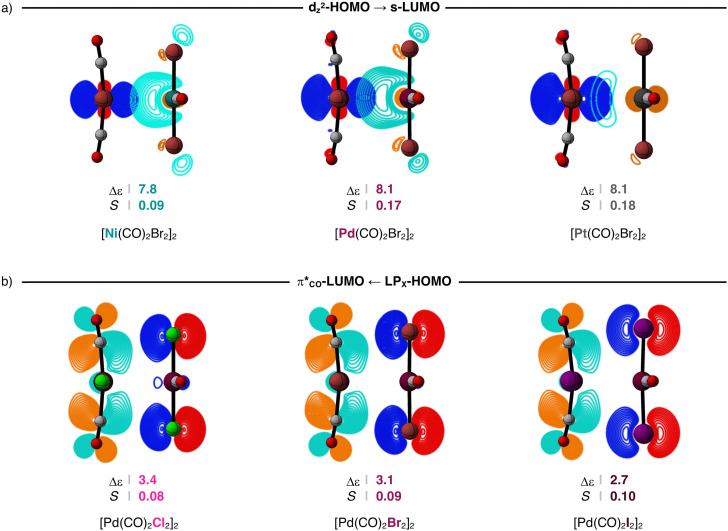
Stabilizing HOMO–LUMO energy gap (in eV) and orbital overlap for (a) the metal–metal and (b) ligand–ligand donor–acceptor interactions for the representative [M(CO)_2_Br_2_]_2_ (M = Ni, Pd, Pt) and [Pd(CO)_2_X_2_]_2_ (X = Cl, Br, I) dimers at a consistent M⋯M bond distance (*r*_M⋯M_ = 3.5 Å; contour plots from 0.9 to 0.04 a.u.). Computed at ZORA-BLYP-D3(BJ)/TZ2P.

The Δ*E*_oi_(*r*_M⋯M_) curves for the [M(CO)_2_X_2_]_2_ dimers become more stabilizing along M = Ni, Pd, Pt because the metal–metal donor–acceptor interactions between the d_*Z*^2^_-type HOMO of one monomer and the s-type LUMO of the other monomer are strengthened along the same series. When the metal center increases in size along Ni, Pd, and Pt, the bond overlap *S* between the d_*Z*^2^_-HOMO and the s-LUMO increases as both orbitals become more diffuse, resulting in larger orbital stabilization and, thus, stronger donor–acceptor interactions along the same series. For example, in the series of [Ni(CO)_2_Br_2_]_2_, [Pd(CO)_2_Br_2_]_2_, and [Pt(CO)_2_Br_2_]_2_ at *r*_M⋯M_ = 3.5, *S* increases from 0.09 to 0.17 to 0.18, as both the d_*Z*^2^_-HOMO and the s-LUMO further extend towards the other metal center (see Fig. 7a, but also the overlap densities in Fig. S2a, ESI†). Consequently, *S*^2^/Δ*ε* times 10^3^ increases from 1.0 to 3.6 to 4.0 along the same series (Table 2).

The Δ*E*_oi_ term in the [M(CO)_2_X_2_]_2_ dimers is not fully dominated by the metal–metal donor–acceptor interactions and is, in part, made by ligand–ligand donor–acceptor interactions. This is confirmed by the trends in the Δ*E*_oi_(*r*_M⋯M_) curves that also become more stabilizing along X = Cl, Br, I (Fig. 6). The bond overlap *S* for the π*_CO_-LUMO–LP_X_-HOMO interaction is, in general, significantly smaller than that of the metal–metal donor–acceptor interactions (Table 2). However, as the X ligands become larger and less electronegative along Cl, Br, and I, the LP_X_-HOMO extends further towards the π*_CO_-LUMO and becomes higher in energy (see Fig. S2b and Table S2 for the orbital energies, ESI†). This results in a larger *S* and a smaller Δ*ε* between the π*_CO_-LUMO and the LP_X_-HOMO. For example, in the series of [Pd(CO)_2_Cl_2_]_2_, [Pd(CO)_2_Br_2_]_2_, and [Pd(CO)_2_I_2_]_2_ at *r*_M⋯M_ = 3.5, *S* increases from 0.08 to 0.09 to 0.10 and Δ*ε* decreases from 3.4 eV to 3.1 eV to 2.7 eV. Consequently, *S*^2^/Δ*ε* times 10^3^ increases from 1.9 to 2.6 to 3.7 along the same series (Table 2).

Since the Δ*E*_oi_ term is not made of one single component, our analyses show that there is an interplay between the metal–metal and ligand–ligand donor–acceptor interactions in the [M(CO)_2_X_2_]_2_ dimers along M = Ni, Pd, Pt and along X = Cl, Br, I. Therefore, to understand the relative importance of the metal–metal and ligand–ligand interactions, we computed the magnitude of their orbital stabilization according to eqn (3) and estimated their relative contribution to the total orbital stabilization (metal–metal + ligand–ligand). The results are collected in Table 2.

When the metal centers are large and the ligands are small and more electronegative, the donor–acceptor metal–metal interactions are strengthened and dominate over the weakened ligand–ligand interactions. For example, for [Pt(CO)_2_Cl_2_]_2_ at *r*_M⋯M_ = 3.5 Å, the metal–metal interactions are up to 67% of the total orbital stabilization. On the other hand, the ligand–ligand donor–acceptor interactions only dominate when the metal center is small and the ligands are large and less electronegative, like in the [Ni(CO)_2_I_2_]_2_ dimer, in which the ligand–ligand donor–acceptor interactions are up to 75% of the total orbital stabilization at *r*_M⋯M_ = 3.5 Å (Table 2).

## 4. Conclusions

The stability of square planar [X_2_(CO)_2_M]⋯[M(CO)_2_X_2_] dimers increases as the metal centers M vary along Ni, Pd, and Pt, and the X ligands vary along Cl, Br, and I. Our quantum chemical bonding analyses show that the dimers are formed due to stabilizing electrostatic interactions on top of stabilizing dispersion interactions promoted by the ligands. Both interactions become more stabilizing as M and X increase in size. In addition, our analyses revealed an overlooked covalent component stemming from metal–metal and ligand–ligand donor–acceptor interactions. These findings emerge from our quantitative Kohn–Sham molecular orbital analyses using dispersion-corrected relativistic density functional theory.

The stability of the studied [M(CO)_2_X_2_]_2_ dimers increases as M varies along Ni, Pd, and Pt as the electron density of one monomer becomes more diffuse around M and more effectively interpenetrates towards the nucleus of M on the other monomer, resulting in a stronger electron–nuclei electrostatic attraction. Larger X ligands further increase the stability of [M(CO)_2_X_2_]_2_ due to a greater dispersion interaction as X increases in size along Cl, Br, and I. The dispersion component in [X_2_(CO)_2_M]⋯[M(CO)_2_X_2_], which is almost insensitive to variations in the metal center, only dominates when M is small, and X is large.

The overlooked covalent component in [X_2_(CO)_2_M]⋯[M(CO)_2_X_2_] comprises the metal–metal donor–acceptor interaction of the *n*d_*z*^2^_-type HOMOs of one monomer with (*n* + 1)s-type LUMOs of the other monomer as well as the ligand–ligand donor–acceptor interaction of the C=O π*-type LUMO on one monomer with the X lone-pair-type HOMO on the other monomer. The metal–metal donor–acceptor interaction dominates when M is large, resulting in a larger stabilizing *n*d_*z*^2^_-HOMO–(*n* + 1)s-LUMO overlap. On the other hand, the ligand–ligand donor–acceptor interactions dominate when M is small and X becomes less electronegative. In this situation, the metal–metal donor–acceptor interaction is weakened, and the ligand–ligand donor–acceptor interaction is favored due to a smaller π*_CO_-LUMO–LP_X_-HOMO energy gap.

## Data availability

The data that support the findings of this study are available in the ESI,† of this article.

## Conflicts of interest

There are no conflicts to declare.

## Supplementary Material

CP-026-D4CP00250D-s001
