# Supplementary material for: The nature of metallophilic interactions in closed-shell d8–d8 metal complexes
Source: Phys Chem Chem Phys. 2024 Jul 18;26(31):20928–36. doi: 10.1039/d4cp00250d (PMC11305097; doi:10.1039/d4cp00250d)
Supplement: CP-026-D4CP00250D-s001 [file CP-026-D4CP00250D-s001.pdf]

Electronic Supplementary Material (ESI) for Physical Chemistry Chemical Physics.
This journal is © the Owner Societies 2024

Supporting Information

for

The Nature of Metallophilic Interactions in Closed-Shell d^8–d^8 Metal Complexes

Lucas de Azevedo Santos,[+a] Timon Wagner,[+a] Klaas Visscher,[a] Jörn Nitsch,[a] F. Matthias Bickelhaupt*[abc] and Célia Fonseca Guerra*[a]

[a] Department of Chemistry and Pharmaceutical Sciences, AIMMS, Vrije Universiteit Amsterdam, De Boelelaan 1108, 1081 HZ Amsterdam, The Netherlands. E-mail: c.fonsecaguerra@vu.nl and f.m.bickelhaupt@vu.nl; *Web*: https://www.theochem.nl

[b] Institute for Molecules and Materials, Radboud University, Heyendaalseweg 135, 6525 AJ Nijmegen, The Netherlands.

[c] Department of Chemical Sciences, University of Johannesburg, Auckland Park, Johannesburg 2006, South Africa.

[+] L.A.S and T.W. contributed equally to this work.

Table of Contents

Complementary Data: $[M(CO_2X_2]_2$ Bonding Mechanism S3

Fig. S1. Attractive overlap between the positively charged nuclei and the negatively charged electron densities (density contours from 0.0001 to 0.5000 a.u.) for representative $[Pd(CO)_2X_2]_2$ dimers (X = Cl, Br, I) at a consistent M•••M bond distance ($r_{M\cdots M}$ = 3.5 Å). Computed at ZORA-BLYP-D3(BJ)/TZ2P.

Fig. S2 Overlap densities for the stabilizing a) metal–metal and b) ligand–ligand donor–acceptor interactions for the representative $[M(CO)_2Br_2]_2$ (M = Ni, Pd, Pt) and $[Pd(CO)_2X_2]_2$ (X = Cl, Br, I) dimers at a consistent M•••M bond distance ($r_{M\cdots M}$ = 3.5 Å; contour plots from 0.009 to 0.0006 a.u.). Computed at ZORA-BLYP-D3(BJ)/TZ2P.

Table S1. HOMO–LUMO orbital overlap (*S*), orbital energies (ε, in eV), and gross Mulliken populations (in electrons) of the metal–metal interaction in $[M(CO)_2X_2]_2$ dimers (M = Ni, Pd, Pt; X = Cl, Br, I) at a consistent M•••M bond distance ($r_{M\cdots M}$ = 3.5 Å). Computed at ZORA-BLYP-D3(BJ)/TZ2P.

Table S2. Donor–acceptor orbital overlap (*S*) orbital energies (ε, in eV), and gross Mulliken populations (in electrons) of the ligand–ligand interaction in $[M(CO)_2X_2]_2$ dimers (M = Ni, Pd, Pt; X = Cl, Br, I) at a consistent M•••M bond distance ($r_{M\cdots M}$ = 3.5 Å). Computed at ZORA-BLYP-D3(BJ)/TZ2P.

Fig. S3. DFT orbitals (isosurfaces at 0.03 a.u.) and orbital energies (in eV) of the $[M(CO)_2X_2]$ monomers in the $[M(CO)_2X_2]_2$ dimers (M = Ni, Pd, Pt; X = Cl, Br, I) at a consistent M•••M bond distance ($r_{M\cdots M}$ = 3.5 Å). Computed at ZORA-BLYP-D3(BJ)/TZ2P.

Potential energy surface **S6**

Table S3. Electronic bond energy of formation of the trans and cis isomers of the $[M(CO)_2X_2]$ monomers[a] and $[M(CO)_2X_2]_2$ dimers[b] (in kcal mol^{-1}). Computed at ZORA-BLYP-D3(BJ)/TZ2P.

Fig. S4. Equilibrium geometries (in Å) and electronic bond energies (in kcal mol^{-1}) of the cis-$[M(CO)_2X_2]_2$ dimers (M = Ni, Pd, Pt; X = Cl, Br, I). Computed at ZORA-BLYP-D3(BJ)/TZ2P.

Fig. S5. Dimerization energies (solid lines; in kcal mol^{-1}) and M•••M bond distance (dashed lines; in Å) as a function of the X–M•••M–X dihedral angle (in deg.) for the $[M(CO)_2X_2]_2$ dimers (M = Ni, Pd, Pt; X = Cl, Br, I). Computed at ZORA-BLYP-D3(BJ)/TZ2P.

Fig. S6. Equilibrium geometries (in Å) and electronic bond energies (in kcal mol^{-1}) of the eclipsed $[M(CO)_2X_2]_2$ dimers (M = Ni, Pd, Pt; X = Cl, Br, I). Computed at ZORA-BLYP-D3(BJ)/TZ2P.

Cartesian coordinates **S8**

Table S4. Cartesian coordinates (in Å), electronic energies (in kcal mol^{-1}), and the number of imaginary vibrational frequencies of the $[M(CO)_2X_2]$ monomers and $[M(CO)_2X_2]_2$ dimers, optimized under the indicated symmetry constraint. Computed at ZORA-BLYP-D3(BJ)/TZ2P.

Table S5. Cartesian coordinates (in Å), electronic energies (in kcal mol^{-1}), and the number of imaginary vibrational frequencies of the cis-$[M(CO)_2X_2]$ monomers and cis-$[M(CO)_2X_2]_2$ dimers, optimized under the indicated symmetry constraint. Computed at ZORA-BLYP-D3(BJ)/QZ4P.

Complementary Data: $[M(CO_2X_2]_2$ Bonding Mechanism

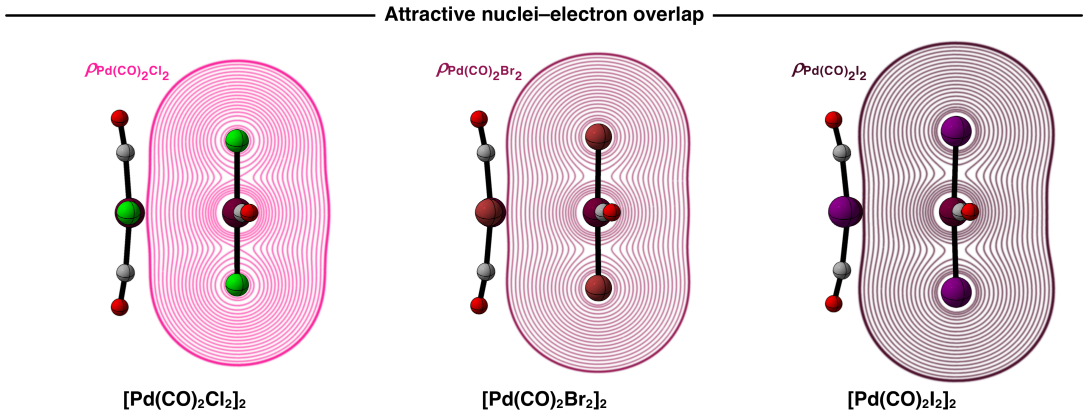

Fig. S1. Attractive overlap between the positively charged nuclei and the negatively charged electron densities (density contours from 0.0001 to 0.5000 a.u.) for representative $[Pd(CO)_2X_2]_2$ dimers (X = Cl, Br, I) at a consistent M•••M bond distance ($r_{M\cdots M}$ = 3.5 Å). Computed at ZORA-BLYP-D3(BJ)/TZ2P.

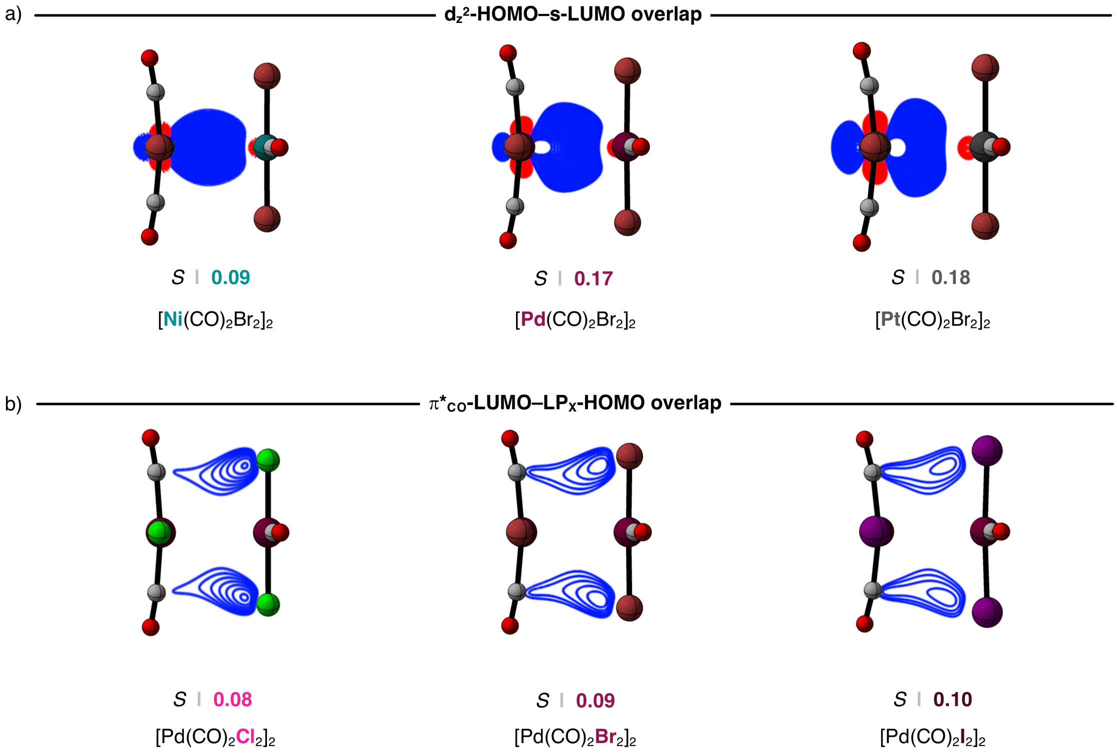

Fig. S2 Overlap densities for the stabilizing a) metal–metal and b) ligand–ligand donor–acceptor interactions for the representative $[M(CO)_2Br_2]_2$ (M = Ni, Pd, Pt) and $[Pd(CO)_2X_2]_2$ (X = Cl, Br, I) dimers at a consistent M•••M bond distance ($r_{M\cdots M}$ = 3.5 Å; contour plots from 0.009 to 0.0006 a.u.). Computed at ZORA-BLYP-D3(BJ)/TZ2P.

Table S1. HOMO–LUMO orbital overlap (*S*), orbital energies (ε, in eV), and gross Mulliken populations (in electrons) of the metal–metal interaction in $[M(CO)_2X_2]_2$ dimers (M = Ni, Pd, Pt; X = Cl, Br, I) at a consistent M•••M bond distance ($r_{M\cdots M}$ = 3.5 Å). Computed at ZORA-BLYP-D3(BJ)/TZ2P.

			d_{z^2}-HOMO		s-LUMO			
**M**	**X**	***S***	**ε**	**Pop.**	**ε**	**Pop.**	**Δε**	**10^3xS^2/Δε**
**Ni**	**Cl**	0.11	–6.9	1.984	1.3	0.007	8.2	1.5
	**Br**	0.09	–6.8	1.982	1.0	0.006	7.8	1.0
	**I**	0.08	–6.7	1.966	0.6	0.005	7.3	0.9
**Pd**	**Cl**	0.18	–7.7	1.975	0.7	0.013	8.4	3.9
	**Br**	0.17	–7.6	1.973	0.5	0.013	8.1	3.6
	**I**	0.16	–7.5	1.971	0.2	0.014	7.7	3.3
**Pt**	**Cl**	0.20	–7.6	1.970	0.6	0.012	8.2	4.9
	**Br**	0.18	–7.6	1.969	0.5	0.008	8.1	4.0
	**I**	0.17	–7.5	1.970	0.3	0.009	7.8	3.7

Table S2. Donor–acceptor orbital overlap (*S*) orbital energies (ε, in eV), and gross Mulliken populations (in electrons) of the ligand–ligand interaction in $[M(CO)_2X_2]_2$ dimers (M = Ni, Pd, Pt; X = Cl, Br, I) at a consistent M•••M bond distance ($r_{M\cdots M}$ = 3.5 Å). Computed at ZORA-BLYP-D3(BJ)/TZ2P.

			LP_X-HOMO		π^*_{CO}-LUMO			
**M**	**X**	***S***	**ε**	**Pop.**	**ε**	**Pop.**	**Δε**	**10^3xS^2/Δε**
**Ni**	**Cl**	0.07	–7.7	1.982	–3.9	0.023	3.8	1.3
	**Br**	0.08	–7.3	1.981	–3.8	0.028	3.5	1.8
	**I**	0.09	–6.8	1.989	–3.7	0.040	3.1	2.6
**Pd**	**Cl**	0.08	–7.4	1.978	–4.0	0.024	3.4	1.9
	**Br**	0.09	–7.0	1.972	–3.9	0.030	3.1	2.6
	**I**	0.10	–6.5	1.962	–3.8	0.040	2.7	3.7
**Pt**	**Cl**	0.09	–7.5	1.972	–4.2	0.038	3.3	2.5
	**Br**	0.10	–7.1	1.968	–4.1	0.047	3.0	3.3
	**I**	0.11	–6.6	1.955	–4.0	0.062	2.6	4.7

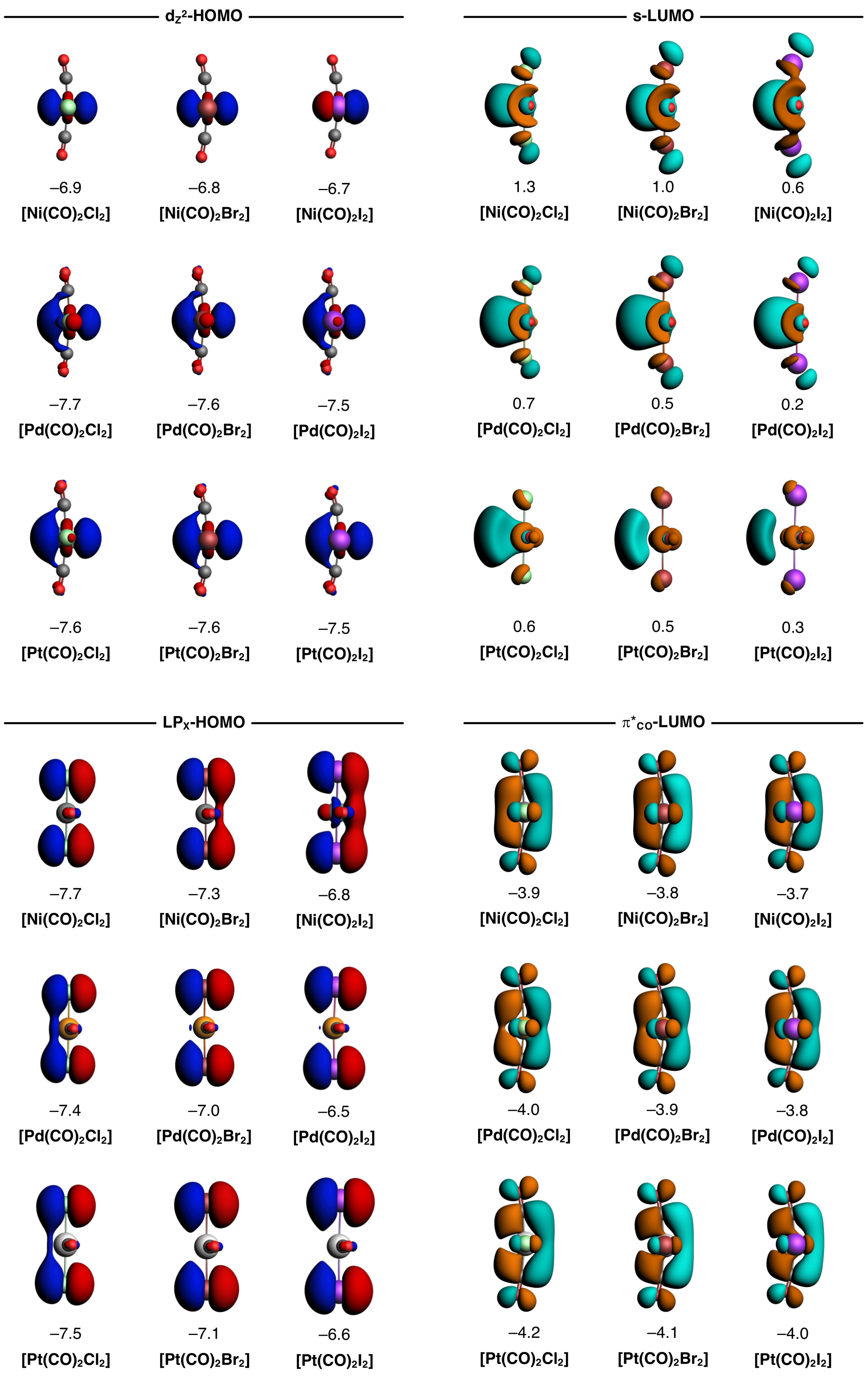

Fig. S3. DFT orbitals (isosurfaces at 0.03 a.u.) and orbital energies (in eV) of the $[M(CO)_2X_2]$ monomers in the $[M(CO)_2X_2]_2$ dimers (M = Ni, Pd, Pt; X = Cl, Br, I) at a consistent M•••M bond distance ($r_{M\cdots M}$ = 3.5 Å). Computed at ZORA-BLYP-D3(BJ)/TZ2P.

Potential energy surface

Table S3. Electronic bond energy of formation of the trans and cis isomers of the $[M(CO)_2X_2]$ monomers[a] and $[M(CO)_2X_2]_2$ dimers[b] (in kcal mol^{-1}). Computed at ZORA-BLYP-D3(BJ)/TZ2P.

M	X	$\Delta \boldsymbol{E}_{\mathbf{mon,trans}}$	$\Delta \boldsymbol{E}_{\mathbf{mon,cis}}$	$\Delta \boldsymbol{E}_{\mathbf{dim,trans}}$	$\Delta \boldsymbol{E}_{\mathbf{dim,cis}}$
**Ni**	**Cl**	–701.8	–700.6	–1416.3	–1416.0
	**Br**	–699.6	–699.9	–1415.1	–1417.6
	**I**	–742.7	–749.0	–1501.9	–1514.3
**Pd**	**Cl**	–691.7	–689.3	–1396.5	–1393.3
	**Br**	–692.1	–690.7	–1400.1	–1399.1
	**I**	–734.1	–737.3	–1485.7	–1491.6
**Pt**	**Cl**	–684.6	–679.9	–1383.1	–1374.4
	**Br**	–686.7	–682.8	–1390.1	–1383.1
	**I**	–729.1	–727.6	–1476.5	–1472.3

[a] $\Delta E_{mon} = E_{mon} - E_{M2+\bullet\bullet} - E_{X-} - E_{CO}$. [b] $\Delta E_{dim} = E_{dim} - 2E_{M2+\bullet\bullet} - 2E_{X-} - 2E_{CO}$.

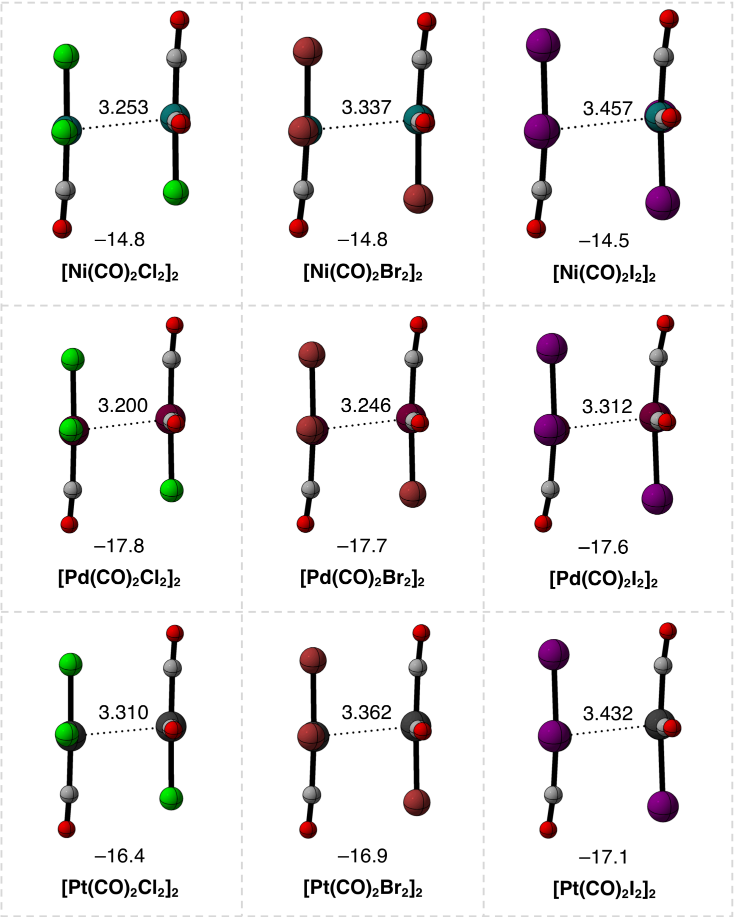

Fig. S4. Equilibrium geometries (in Å) and electronic bond energies (in kcal mol^{-1}) of the cis-$[M(CO)_2X_2]_2$ dimers (M = Ni, Pd, Pt; X = Cl, Br, I). Computed at ZORA-BLYP-D3(BJ)/TZ2P.

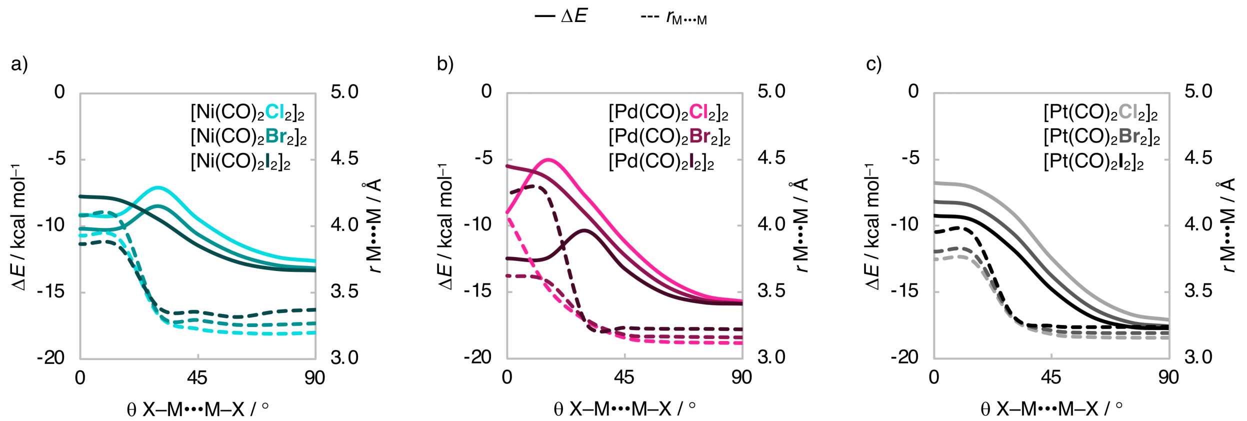

Fig. S5. Dimerization energies (solid lines; in kcal mol^{-1}) and M•••M bond distance (dashed lines; in Å) as a function of the X–M•••M–X dihedral angle (in deg.) for the $[M(CO)_2X_2]_2$ dimers (M = Ni, Pd, Pt; X = Cl, Br, I). Computed at ZORA-BLYP-D3(BJ)/TZ2P.

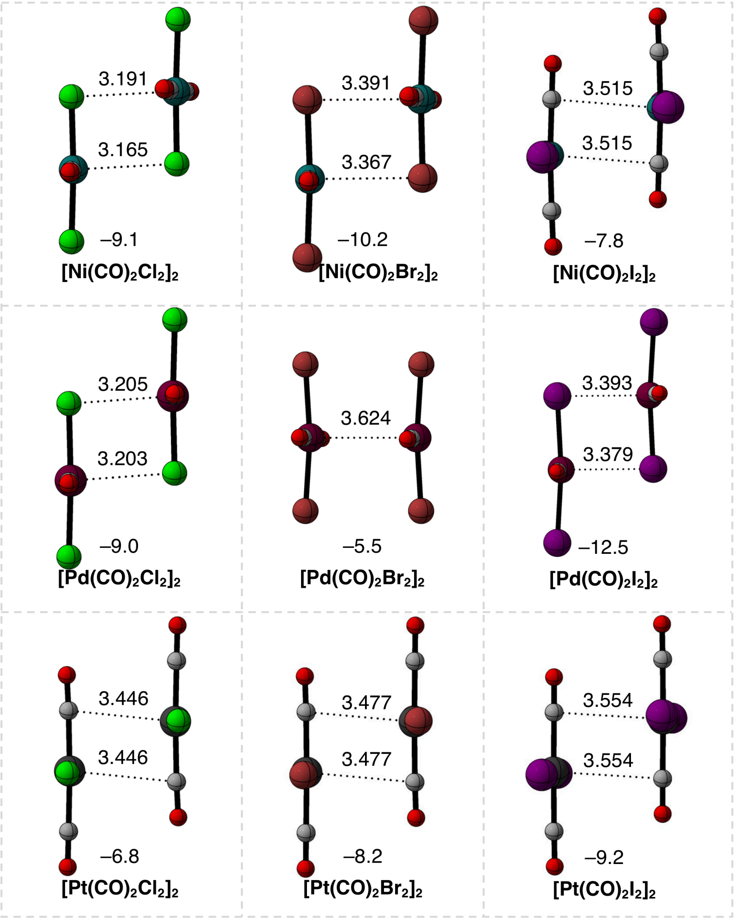

Fig. S6. Equilibrium geometries (in Å) and electronic bond energies (in kcal mol^{-1}) of the eclipsed $[M(CO)_2X_2]_2$ dimers (M = Ni, Pd, Pt; X = Cl, Br, I). Computed at ZORA-BLYP-D3(BJ)/TZ2P.

Cartesian coordinates

Table S4. Cartesian coordinates (in Å), electronic energies (in kcal mol^{-1}), and the number of imaginary vibrational frequencies of the [M(CO)$_2$X$_2$] monomers and [M(CO)$_2$X$_2$]$_2$ dimers, optimized under the indicated symmetry constraint. Computed at ZORA-BLYP-D3(BJ)/QZ4P.

[Ni(CO)$_2$Cl$_2$]
***E* = -921.60**
N$_{imag}$ = 0
Symmetry = *D*$_{2H}$

Ni	0.00000000	0.00000000	0.00000000
Cl	0.00000000	0.00000000	-2.18618472
Cl	0.00000000	0.00000000	2.18618472
C	0.00000000	-1.81576801	0.00000000
O	0.00000000	-2.95453787	0.00000000
C	0.00000000	1.81576801	0.00000000
O	0.00000000	2.95453787	0.00000000

[Ni(CO)$_2$Br$_2$]
***E* = -904.62**
N$_{imag}$ = 0
Symmetry = *D*$_{2H}$

Ni	0.00000000	-0.00000000	0.00000000
Br	0.00000000	-0.00000000	2.33094664
Br	0.00000000	-0.00000000	-2.33094664
C	0.00000000	1.80780530	0.00000000
O	0.00000000	2.94845366	0.00000000
C	0.00000000	-1.80780530	0.00000000
O	0.00000000	-2.94845366	0.00000000

[Ni(CO)$_2$I$_2$]
***E* = -886.70**
N$_{imag}$ = 0
Symmetry = *D*$_{2H}$

Ni	0.00000000	0.00000000	0.00000000
I	0.00000000	0.00000000	2.52435600
I	-0.00000000	0.00000000	-2.52435600
C	-0.00000000	1.79994400	0.00000000
O	-0.00000000	2.94344300	0.00000000
C	0.00000000	-1.79994400	0.00000000
O	0.00000000	-2.94344300	0.00000000

[Pd(CO)$_2$Cl$_2$]
***E* = -872.20**
N$_{imag}$ = 0
Symmetry = *D*$_{2H}$

Pd	0.00000000	0.00000000	0.00000000
Cl	0.00000000	2.34672510	0.00000000
Cl	0.00000000	-2.34672510	0.00000000
C	1.96381718	0.00000000	0.00000000
O	3.09969614	0.00000000	0.00000000
C	-1.96381718	0.00000000	0.00000000
O	-3.09969614	0.00000000	0.00000000

[Pd(CO)$_2$Br$_2$]
***E* = -857.85**
N$_{imag}$ = 0
Symmetry = *D*$_{2H}$

Pd	0.00000000	0.00000000	0.00000000
Br	0.00000000	0.00000000	-2.48592100
Br	0.00000000	0.00000000	2.48592100
C	0.00000000	1.95848900	0.00000000
O	0.00000000	3.09563050	0.00000000
C	0.00000000	-1.95848900	0.00000000
O	0.00000000	-3.09563050	0.00000000

[Pd(CO)$_2$I$_2$]
***E* = -841.61**
N$_{imag}$ = 0
Symmetry = *D*$_{2H}$

Pd	0.00000000	0.00000000	0.00000000
I	-0.00000000	0.00000000	2.67312100
I	0.00000000	0.00000000	-2.67312100
C	0.00000000	1.95399400	0.00000000
O	-0.00000000	3.09312400	0.00000000
C	-0.00000000	-1.95399400	0.00000000
O	0.00000000	-3.09312400	0.00000000

$[Pt(CO)_2Cl_2]$
***E* = -928.79**
N_{imag} = 0
Symmetry = D_{2H}

Pt	0.00000000	0.00000000	0.00000000
Cl	0.00000000	0.00000000	-2.36397306
Cl	0.00000000	0.00000000	2.36397306
C	0.00000000	-1.93943755	0.00000000
O	0.00000000	-3.07763508	0.00000000
C	0.00000000	1.93943755	0.00000000
O	0.00000000	3.07763508	0.00000000

$[Pt(CO)_2Br_2]$
***E* = -913.65**
N_{imag} = 0
Symmetry = D_{2H}

Pt	0.00000000	0.00000000	0.00000000
Br	0.00000000	0.00000000	2.50487500
Br	-0.00000000	0.00000000	-2.50487500
C	-0.00000000	-1.93582300	0.00000000
O	-0.00000000	-3.07522700	0.00000000
C	0.00000000	1.93582300	0.00000000
O	0.00000000	3.07522700	0.00000000

$[Pt(CO)_2I_2]$
***E* = -897.80**
N_{imag} = 0
Symmetry = D_{2H}

Pt	0.00000000	0.00000000	0.00000000
I	0.00000000	0.00000000	-2.69226200
I	-0.00000000	0.00000000	2.69226200
C	-0.00000000	-1.93263400	0.00000000
O	-0.00000000	-3.07396900	0.00000000
C	0.00000000	1.93263400	0.00000000
O	0.00000000	3.07396900	0.00000000

$[Ni(CO)_2Cl_2]_2$
***E* = -1855.79**
N_{imag} = 0
Symmetry = C_S

Ni	-1.59960000	0.00000000	0.00000000
Cl	-1.59720000	-0.00200000	-2.19640000
Cl	-1.59410000	0.00000000	2.19520000
C	-1.72700000	-1.81890000	0.00000000
O	-1.89180000	-2.94470000	0.00100000
C	-1.72970000	1.81760000	-0.00160000
O	-1.89660000	2.94310000	-0.00210000
Ni	1.59960000	0.00000000	0.00000000
Cl	1.59750000	-2.19630000	0.00000000
Cl	1.59330000	2.19530000	-0.00200000
C	1.72750000	-0.00160000	-1.81900000
O	1.89240000	-0.00180000	-2.94480000
C	1.72890000	-0.00030000	1.81760000
O	1.89470000	-0.00060000	2.94320000

$[Ni(CO)_2Br_2]_2$
***E* = -1822.34**
N_{imag} = 0
Symmetry = C_S

Ni	-1.63460000	0.00000000	0.00000000
Br	-1.64210000	0.00000000	2.34110000
Br	-1.64210000	0.00000000	-2.34110000
C	-1.79090000	1.80760000	-0.00000000
O	-1.98680000	2.93050000	-0.00000000
C	-1.79080000	-1.80760000	0.00000000
O	-1.98660000	-2.93050000	0.00000000
Ni	1.63460000	0.00000000	0.00000000
Br	1.64200000	2.34110000	-0.00000000
Br	1.64220000	-2.34110000	0.00000000
C	1.79080000	-0.00000000	1.80760000
O	1.98670000	0.00000000	2.93050000
C	1.79080000	0.00010000	-1.80760000
O	1.98670000	0.00020000	-2.93050000

$[Ni(CO)_2I_2]_2$
***E* = -1787.20**
N_{imag} = 0
Symmetry = C_S

Ni	-1.68550000	-0.00000000	0.00000000
I	-1.71010000	0.00180000	2.53440000
I	-1.71250000	-0.00180000	-2.53430000
C	-1.88370000	1.79430000	-0.00130000
O	-2.11610000	2.91350000	-0.00200000
C	-1.88090000	-1.79490000	0.00130000
O	-2.11150000	-2.91440000	0.00220000
Ni	1.68550000	0.00000000	0.00000000
I	1.70990000	2.53490000	-0.00000000
I	1.71360000	-2.53380000	0.00180000
C	1.88380000	0.00190000	1.79520000
O	2.11680000	0.00200000	2.91430000
C	1.88200000	-0.00060000	-1.79380000
O	2.11380000	-0.00110000	-2.91310000

$[Pd(CO)_2Cl_2]_2$
***E* = -1760.20**
N_{imag} = 0
Symmetry = *C*s

Pd	-1.55860000	-0.00000000	0.00000000
Cl	-1.55760000	-0.00050000	2.34990000
Cl	-1.56530000	0.00000000	-2.34980000
C	-1.69940000	1.96420000	-0.00030000
O	-1.89030000	3.08320000	0.00010000
C	-1.69800000	-1.96470000	0.00030000
O	-1.88870000	-3.08370000	0.00040000
Pd	1.55860000	0.00000000	0.00000000
Cl	1.55960000	2.34990000	-0.00000000
Cl	1.56150000	-2.34980000	0.00000000
C	1.69990000	-0.00000000	1.96450000
O	1.89110000	0.00020000	3.08340000
C	1.69480000	0.00140000	-1.96460000
O	1.88230000	0.00330000	-3.08420000

$[Pd(CO)_2Br_2]_2$
***E* = -1731.60**
N_{imag} = 0
Symmetry = *C*s

Pd	-1.57980000	0.00000000	0.00000000
Br	-1.61070000	-0.00010000	-2.48870000
Br	-1.61060000	0.00010000	2.48880000
C	-1.74800000	1.95650000	0.00000000
O	-1.97720000	3.06980000	0.00010000
C	-1.74810000	-1.95650000	-0.00000000
O	-1.97720000	-3.06980000	-0.00010000
Pd	1.57980000	0.00000000	0.00000000
Br	1.61070000	2.48870000	0.00000000
Br	1.61060000	-2.48870000	0.00000000
C	1.74800000	0.00000000	-1.95650000
O	1.97720000	0.00000000	-3.06980000
C	1.74810000	0.00000000	1.95650000
O	1.97720000	-0.00000000	3.06980000

$[Pd(CO)_2I_2]_2$
***E* = -1699.82**
N_{imag} = 0
Symmetry = *C*s

Pd	-1.61020000	0.00000000	0.00000000
I	-1.68510000	0.00000000	-2.67400000
I	-1.69860000	-0.00000000	2.67360000
C	-1.81820000	-1.94760000	-0.00000000
O	-2.09040000	-3.05350000	-0.00040000
C	-1.82040000	1.94740000	0.00000000
O	-2.09430000	3.05300000	-0.00070000
Pd	1.61020000	0.00000000	-0.00000000
I	1.69240000	-2.67370000	-0.00000000
I	1.68870000	2.67390000	0.00000000
C	1.82220000	0.00000000	-1.94730000
O	2.09660000	0.00020000	-3.05270000
C	1.81430000	0.00010000	1.94790000
O	2.08390000	-0.00000000	3.05450000

$[Pt(CO)_2Cl_2]_2$
***E* = -1874.68**
N_{imag} = 0
Symmetry = *C*s

Pt	-1.57860000	0.00000000	0.00000000
Cl	-1.55590000	-0.00000000	-2.36860000
Cl	-1.55480000	0.00000000	2.36860000
C	-1.74340000	-1.93770000	0.00000000
O	-1.94500000	-3.05750000	0.00020000
C	-1.74430000	1.93770000	-0.00000000
O	-1.94640000	3.05740000	-0.00020000
Pt	1.57860000	0.00000000	0.00000000
Cl	1.55600000	-2.36860000	0.00000000
Cl	1.55470000	2.36860000	-0.00000000
C	1.74400000	-0.00000000	-1.93770000
O	1.94610000	0.00010000	-3.05740000
C	1.74370000	-0.00060000	1.93780000
O	1.94550000	-0.00140000	3.05750000

$[Pt(CO)_2Br_2]_2$
***E* = -1844.90**
N_{imag} = 0
Symmetry = *C*s

Pt	-1.59640000	0.00000000	-0.00000000
Br	-1.59170000	0.00000000	2.50990000
Br	-1.59170000	-0.00000000	-2.50990000
C	-1.78870000	1.93080000	0.00010000
O	-2.02960000	3.04430000	0.00020000
C	-1.78870000	-1.93080000	-0.00010000
O	-2.02960000	-3.04430000	-0.00020000
Pt	1.59640000	0.00000000	0.00000000
Br	1.59170000	2.50990000	0.00000000
Br	1.59170000	-2.50990000	-0.00000000
C	1.78870000	-0.00010000	1.93080000
O	2.02960000	-0.00020000	3.04430000
C	1.78870000	0.00010000	-1.93080000
O	2.02960000	0.00020000	-3.04430000

$[Pt(CO)_2I_2]_2$
***E* = -1813.90**
N_{imag} = 0
Symmetry = C_s

Pt	-1.61970000	-0.00010000	0.00010000
I	-1.65890000	-0.00010000	-2.69620000
I	-1.65460000	-0.00010000	2.69650000
C	-1.84970000	1.92240000	0.00010000
O	-2.13740000	3.02710000	0.00000000
C	-1.84960000	-1.92270000	0.00010000
O	-2.13750000	-3.02740000	-0.00020000
Pt	1.61980000	-0.00010000	0.00010000
I	1.65690000	2.69620000	0.00010000
I	1.65660000	-2.69640000	0.00010000
C	1.84800000	-0.00010000	-1.92250000
O	2.13450000	0.00000000	-3.02760000
C	1.85130000	0.00030000	1.92250000
O	2.14040000	0.00100000	3.02690000

CO
***E* = -333.20**
N_{imag} = 0
Symmetry = $C_{\infty v}$

C	0.00000000	0.00000000	-0.56836579
O	0.00000000	0.00000000	0.56836579

Table S5. Cartesian coordinates (in Å), electronic energies (in kcal mol^{-1}), and the number of imaginary vibrational frequencies of the cis-[M(CO)$_2$X$_2$] monomers and cis-[M(CO)$_2$X$_2$]$_2$ dimers, optimized under the indicated symmetry constraint. Computed at ZORA-BLYP-D3(BJ)/QZ4P.

[Ni(CO)$_2$Cl$_2$]
***E* = -920.40**
N$_{imag}$ = 0
Symmetry = *C*$_{2v}$

Ni	0.00000000	0.00000000	-0.49144243
O	0.00000000	-2.24639295	1.44052651
Cl	0.00000000	1.63042245	-1.89687515
C	0.00000000	1.37296407	0.70206985
O	0.00000000	2.24639295	1.44052651
Cl	0.00000000	-1.63042245	-1.89687515
C	0.00000000	-1.37296407	0.70206985

[Ni(CO)$_2$Br$_2$]
***E* = -902.19**
N$_{imag}$ = 0
Symmetry = *C*$_{2v}$

Ni	0.00000000	0.00000000	0.42680872
O	0.00000000	2.22218707	-1.47852235
Br	0.00000000	-1.75374291	1.97471144
C	0.00000000	-1.37307993	-0.70959345
O	0.00000000	-2.22218707	-1.47852235
Br	0.00000000	1.75374291	1.97471144
C	0.00000000	1.37307993	-0.70959345

[Ni(CO)$_2$I$_2$]
***E* = -881.97**
N$_{imag}$ = 0
Symmetry = *C*$_{2v}$

Ni	0.00000000	0.00000000	-0.40518081
O	0.00000000	-2.18346579	1.54758229
I	0.00000000	1.90959104	-2.08657135
C	0.00000000	1.36646010	0.74157946
O	0.00000000	2.18346579	1.54758229
I	0.00000000	-1.90959104	-2.08657135
C	0.00000000	-1.36646010	0.74157946

[Pd(CO)$_2$Cl$_2$]
***E* = -872.51**
N$_{imag}$ = 0
Symmetry = *C*$_{2v}$

Pd	0.00000000	0.00000000	-0.50022757
O	0.00000000	-2.39504314	1.49640036
Cl	0.00000000	1.69177533	-2.03862293
C	0.00000000	1.49787588	0.79233635
O	0.00000000	2.39504314	1.49640036
Cl	0.00000000	-1.69177533	-2.03862293
C	0.00000000	-1.49787588	0.79233635

[Pd(CO)$_2$Br$_2$]
***E* = -856.47**
N$_{imag}$ = 0
Symmetry = *C*$_{2v}$

Pd	0.00000000	0.00000000	-0.42544474
O	0.00000000	-2.39897560	1.51842484
Br	0.00000000	1.80927434	-2.10524878
C	0.00000000	1.51220718	0.79954631
O	0.00000000	2.39897560	1.51842484
Br	0.00000000	-1.80927434	-2.10524878
C	0.00000000	-1.51220718	0.79954631

[Pd(CO)$_2$I$_2$]
***E* = -837.62**
N$_{imag}$ = 0
Symmetry = *C*$_{2v}$

Pd	0.00000000	0.00000000	-0.39794745
O	0.00000000	-2.38864976	1.57649547
I	0.00000000	1.95579967	-2.20705941
C	0.00000000	1.52341468	0.82953766
O	0.00000000	2.38864976	1.57649547
I	0.00000000	-1.95579967	-2.20705941
C	0.00000000	-1.52341468	0.82953766

[Pt(CO)$_2$Cl$_2$]
***E* = -935.37**
N$_{imag}$ = 0
Symmetry = *C*$_{2v}$

Pt	0.00000000	0.00000000	0.46096117
O	0.00000000	2.30885728	-1.53278370
Cl	0.00000000	-1.66247718	2.09410064
C	0.00000000	-1.43669794	-0.79179753
O	0.00000000	-2.30885728	-1.53278370
Cl	0.00000000	1.66247718	2.09410064
C	0.00000000	1.43669794	-0.79179753

[Pt(CO)$_2$Br$_2$]
***E* = -916.90**
N$_{imag}$ = 0
Symmetry = *C*$_{2v}$

Pt	0.00000000	0.00000000	-0.42944371
O	0.00000000	-2.32332369	1.55296838
Br	0.00000000	1.78479075	-2.14671417
C	0.00000000	1.45310749	0.80846765
O	0.00000000	2.32332369	1.55296838
Br	0.00000000	-1.78479075	-2.14671417
C	0.00000000	-1.45310749	0.80846765

$[Pt(CO)_2I_2]$
***E* = -896.28**
N_{imag} = 0
Symmetry = C_{2v}

Pt	0.00000000	0.00000000	-0.39608380
O	0.00000000	-2.32614204	1.59663495
I	0.00000000	1.94480925	-2.23455213
C	0.00000000	1.46841793	0.83595909
O	0.00000000	2.32614204	1.59663495
I	0.00000000	-1.94480925	-2.23455213
C	0.00000000	-1.46841793	0.83595909

$[Ni(CO)_2Cl_2]_2$
***E* = -1855.55**
N_{imag} = 0
Symmetry = C_1

Ni	-1.61716100	-0.09513500	-0.15535100
O	-1.84236600	0.13186400	-3.07105700
Cl	-1.56839200	-0.18717100	2.04307100
C	-1.71273700	-1.88010200	-0.01497100
O	-1.83927100	-3.01087600	0.07309800
Cl	-1.57151700	2.10325400	-0.24829900
C	-1.71477300	0.04433100	-1.94034900
Ni	1.61659800	0.15519400	0.09263600
Cl	1.57036800	-2.04323800	0.18583700
O	1.83941500	3.07093000	-0.13622100
Cl	1.56850300	0.24666500	-2.10577400
C	1.71275500	1.94018200	-0.04794600
C	1.71367800	0.01641100	1.87769500
O	1.84112300	-0.07048300	3.00846700

$[Ni(CO)_2Br_2]_2$
***E* = -1819.15**
N_{imag} = 0
Symmetry = C_1

Ni	-1.65529300	-0.12159800	-0.18179500
O	-1.90210800	0.08225300	-3.09673700
Br	-1.65023100	-0.23505500	2.16727800
C	-1.75631300	-1.90388000	-0.03409800
O	-1.90012300	-3.03663700	0.02323400
Br	-1.65172900	2.22678000	-0.29519200
C	-1.75757300	0.02525600	-1.96406200
Ni	1.65467100	0.18186500	0.11909900
Br	1.65137900	-2.16679800	0.23397600
O	1.89940300	3.09685000	-0.08726700
Br	1.64938800	0.29395900	-2.22968200
C	1.75561200	1.96412600	-0.02932600
C	1.75718600	0.03558600	1.90136400
O	1.90195200	-0.02088000	3.03404400

$[Ni(CO)_2I_2]_2$
***E* = -1778.47**
N_{imag} = 0
Symmetry = C_1

Ni	-1.70786900	-0.15757400	-0.22381900
O	-1.99759900	-0.01001500	-3.13960400
I	-1.75405800	-0.27974800	2.32973300
C	-1.82259500	-1.93983900	-0.07896300
O	-1.98777700	-3.07361500	-0.06457200
I	-1.76654700	2.39526400	-0.35667200
C	-1.82908000	-0.01930400	-2.00624300
Ni	1.70748900	0.21842000	0.16048200
I	1.76431700	-2.33468400	0.28734600
O	1.98533900	3.13504800	0.00807800
I	1.75477600	0.34658100	-2.39289100
C	1.82150100	2.00105800	0.01962200
C	1.82944500	0.07616300	1.94257100
O	1.99888200	0.06406900	3.07576800

[Pd(CO)$_2$Cl$_2$]$_2$
***E* = -1762.78**
N$_{imag}$ = 0
Symmetry = *C*$_1$

Pd	-1.59412500	-0.06178500	-0.13185100
O	-1.89664300	0.19604100	-3.19192100
Cl	-1.50203300	-0.10283200	2.20047100
Cl	-1.50390600	2.27028500	-0.17942300
O	1.89700900	-0.13439100	3.12018100
C	-1.73338200	-2.00043000	0.01921900
O	-1.89543900	-3.12111500	0.13515400
Pd	1.59411400	0.13264900	0.06100700
O	1.89507300	3.19288100	-0.19670200
Cl	1.50310900	-2.19972500	0.10191800
Cl	1.50305800	0.18044800	-2.27101900
C	1.73437800	-0.01846600	1.99957900
C	-1.73439200	0.08387900	-2.07088700
C	1.73343600	2.07175700	-0.08456000

[Pd(CO)$_2$Br$_2$]$_2$
***E* = -1730.62**
N$_{imag}$ = 0
Symmetry = *C*$_1$

Pd	-1.61301900	-0.09021300	-0.16077300
O	-1.96824700	0.17034800	-3.21990200
Br	-1.56793900	-0.14391800	2.31457800
Br	-1.57012700	2.38498400	-0.22429400
O	1.96959700	-0.11316300	3.14709700
C	-1.76703400	-2.03126900	0.00873700
O	-1.96806200	-3.14809000	0.11394000
Pd	1.61307900	0.16165000	0.08939900
O	1.96692900	3.22093700	-0.17108800
Br	1.56893200	-2.31373900	0.14290200
Br	1.56899800	0.22534400	-2.38575900
C	1.76798600	-0.00794600	2.03038300
C	-1.76753400	0.07083100	-2.10249200
C	1.76670100	2.10344000	-0.07156500

[Pd(CO)$_2$I$_2$]$_2$
***E* = -1692.83**
N$_{imag}$ = 0
Symmetry = *C*$_1$

Pd	-1.64007400	-0.12632900	-0.19551300
O	-2.07693100	0.12136500	-3.25614000
I	-1.66639000	-0.19127500	2.47324600
I	-1.66800700	2.54263100	-0.26056400
O	2.07718700	-0.05186000	3.18639200
C	-1.82177300	-2.07682300	-0.02100800
O	-2.07561300	-3.18736400	0.05266900
Pd	1.64012300	0.19631800	0.12557400
O	2.07540500	3.25717200	-0.12212400
I	1.66768800	-2.47243200	0.19032700
I	1.66680600	0.26155800	-2.54339600
C	1.82279600	0.02177200	2.07597200
C	-1.82274800	0.04789000	-2.14566700
C	1.82179100	2.14657000	-0.04860400

[Pt(CO)$_2$Cl$_2$]$_2$
***E* = -1887.12**
N$_{imag}$ = 0
Symmetry = *C*$_1$

Pt	-1.65085400	-0.04788700	-0.11804600
O	-1.89170900	0.14406200	-3.15452000
Cl	-1.56354200	-0.05742400	2.22193900
Cl	-1.56495000	2.29201800	-0.13447300
O	1.89205800	-0.08276900	3.08296000
C	-1.76791200	-1.95162300	-0.00158600
O	-1.89054100	-3.08387400	0.08353700
Pt	1.65092200	0.11889000	0.04709100
O	1.89030900	3.15548200	-0.14476800
Cl	1.56449900	-2.22113700	0.05651600
Cl	1.56399000	0.13543000	-2.29276700
C	1.76886500	0.00236100	1.95077100
C	-1.76880500	0.06272100	-2.02204500
C	1.76793100	2.02294800	-0.06344400

[Pt(CO)$_2$Br$_2$]$_2$
***E* = -1850.73**
N$_{imag}$ = 0
Symmetry = *C*$_1$

Pt	-1.67343700	-0.07690700	-0.14697900
O	-1.94955600	0.13155400	-3.18271100
Br	-1.62168400	-0.10895700	2.33887100
Br	-1.62333500	2.40878100	-0.18650100
O	1.95029400	-0.07041000	3.11111600
C	-1.79824900	-1.98187500	-0.01083700
O	-1.94882900	-3.11204400	0.07119900
Pt	1.67345400	0.14783100	0.07611400
O	1.94818600	3.18369200	-0.13229400
Br	1.62289900	-2.33805100	0.10803200
Br	1.62218800	0.18746200	-2.40954100
C	1.79917800	0.01162500	1.98101900
C	-1.79885800	0.05329500	-2.05229000
C	1.79801100	2.05320200	-0.05403300

[Pt(CO)$_2$I$_2$]$_2$
***E* = -1809.69**
N$_{imag}$ = 0
Symmetry = *C*$_1$

Pt	-1.70333700	-0.11305500	-0.18354800
O	-2.04257300	0.08913100	-3.22175600
I	-1.71310400	-0.16584400	2.49921600
I	-1.71485400	2.56931700	-0.24693900
O	2.04369000	-0.03166000	3.14957600
C	-1.84810100	-2.02423300	-0.03186600
O	-2.04222400	-3.15052600	0.03250900
Pt	1.70338500	0.18438500	0.11226200
O	2.04114300	3.22276100	-0.08990700
I	1.71435300	-2.49838200	0.16490000
I	1.71369800	0.24793500	-2.57010600
C	1.84905500	0.03267900	2.02337000
C	-1.84870100	0.03066900	-2.09511000
C	1.84782900	2.09601900	-0.03143700
